# Synovial tissues concentrate secreted APRIL

**DOI:** 10.1186/ar2817

**Published:** 2009-09-29

**Authors:** Cem Gabay, Veit Krenn, Carine Bosshard, Christian Alexander Seemayer, Carlo Chizzolini, Bertrand Huard

**Affiliations:** 1 Division of Rheumatology, University Hospitals, 4 rue Gabrielle Perret-Gentil, Geneva, CH 1211, Switzerland; 2 Department of Pathology-Immunology, Faculty of Medicine, 1 rue Michel Servet, Geneva, CH 1211, Switzerland; 3 Cytology and Molecular Diagnostic, Histology Center, Max Planck Street, Trier D-54296, Germany; 4 Division of Hematology, 4 rue Gabrielle Perret-Gentil, Geneva, CH 1211, Switzerland; 5 Department of Pathology, Faculty of Medicine, 1 rue Michel Servet, Geneva, CH 1211, Switzerland; 6 Current address: Rheumatology, University Hospitals, 46 rue du Bugnon, Lausanne, CH 1011, Switzerland; 7 Division of Immunology and Allergy, 4 rue Gabrielle Perret-Gentil, Geneva, CH 1211, Switzerland

## Abstract

**Introduction:**

A proliferation-inducing ligand (APRIL) from the TNF family, owing to its role in the generation and survival of plasma cells (PCs), is currently targeted for rheumatoid arthritis (RA) treatment. However, little is known about APRIL expression in RA lesions, hampering our understanding of the way APRIL may modulate this autoimmune disease.

**Methods:**

We performed immunological staining of human normal, non-RA and RA synovial tissues with a pair of antibodies specifically recognizing APRIL-producing cells and secreted APRIL.

**Results:**

We detected significant amounts of secreted APRIL in normal synovium mostly concentrated around blood vessels and at the lining layer, but no cells producing APRIL. Meanwhile, we observed that blood neutrophils constitutively secrete APRIL, indicating that blood APRIL may diffuse into the synovium via its fenestrated vessels. Synovium from non-RA and RA patients retained similarly secreted APRIL, but in this case APRIL-producing cells, including neutrophils and macrophages, were present in the tissue. Notably, PCs - when present in RA synovium - accumulated in areas of APRIL retention, spreading from blood vessels towards the lining layer.

**Conclusions:**

PCs accumulate in synovial zones rich in secreted APRIL, consistent with a pro-survival role of APRIL for PCs in RA. The concentration of APRIL by normal synovium indicates that this tissue may constitute a proper environment for PCs even before RA onset.

## Introduction

A proliferation-inducing ligand (APRIL, TNFSF13) is one of the latest members cloned from the TNF superfamily [1]. APRIL modulates late steps of humoral immune responses by inducing immunoglobulin switch [2-5], and by transmitting a survival signal into plasmablast/plasma cells (PCs) [6-8]. In cancer patients, APRIL promotes selectively the development of chronic lymphocytic leukemia [9] and diffuse large B-cell lymphoma [10]. This selectivity is consistent with the restricted expression of APRIL signaling receptors - the transmembrane activator, calcium modulator and cyclophilin ligand interactor (TACI, TNFSFR13), and the B-cell maturation antigen (BCMA, TNFSFR17) - to specific B-cell differentiation stages [11]. In addition to TACI and BCMA, heparan sulfate proteoglycans (HSPG) bind APRIL [12,13] and TACI [14].

APRIL is also implicated in autoimmune pathologies, particularly rheumatic diseases [15]. Use of a soluble form of TACI that antagonizes both APRIL and the closely related B-cell activation factor from the TNF family (BAFF, TNFSF13B) [16] ameliorated rheumatoid arthritis (RA) in mouse models [17-19]. A subsequent phase I clinical trial with soluble TACI in RA patients showed a decrease in levels of rheumatoid factor and antibodies against citrullinated proteins in treated patients [20,21], indicating promising perspectives for such a therapeutic approach. Expression of BAFF in RA lesions is well characterized, with a wide expression in B cells, T cells, fibroblast-like synoviocytes [22] and monocyte/macrophages [19]. In contrast, APRIL expression appears much more restricted, since only CD83^+ ^dendritic cells [19] and fibroblast-like synoviocytes [23] have been reported to produce APRIL in RA lesions.

Ectopic germinal centers (GCs) with PC generation are present in more than 10% of RA patients [24]. Knowing the role of APRIL in humoral immunity, we studied APRIL expression in RA lesions, with particular attention to lesions with GCs, and compared this expression with normal and non-RA synovium samples. We performed the present study with a pair of well-characterized antibodies selectively recognizing APRIL-producing cells and secreted APRIL in tissues [10]. The pattern of expression for APRIL observed here is consistent with a pro-survival role of APRIL for synovial PCs.

## Materials and methods

### Patients

Synovial biopsies were obtained from the Geneva School of Medicine and from the Charité Universitätsmedizin in agreement with local ethics committees and patients' informed consent upon knee arthroscopy of patients with active disease. Clinical diagnosis was performed for psoriatic arthritis according to Moll and Wright [25], and for RA according to the classification criteria and of the American College of Rheumatology [26]. The presence of rheumatoid factor of IgM serotype or IgA isotype defined seropositivity. We defined GCs by a characteristic histomorphology and the presence of CD23^+ ^follicular dendritic cells [27].

The patient demography is presented in Table 1. Normal synovium samples were obtained during autopsies. Non-inflamed tonsils were obtained from patients who underwent surgery for snoring problems.

**Table 1:** Patient demography

Rheumatoid arthritis	
Sex	8 males 3 females
Age (years)	43 (30 to 78)
Disease duration (years)	3.8 (1 to 7)
Seropositivity	9
With germinal center	3
Treatment	
Nonsteroidal anti-inflammatory drug	4
Methothrexate	3
Methothrexate + prednisolone	3
Lefluonimide + prednisone	1
Non-rheumatoid arthritis	
Psoriatic arthritis	
Sex	2 males 3 females
Age (years)	53 (45 to 80)
Disease duration (years)	10.3 (5 to 12)
Treatment	
Methothrexate	3
Sulfasalazin	2
Lyme	
Sex	Male
Age (years)	55
Disease duration (years)	3
Treatment	
Nonsteroidal anti-inflammatory drug	
Gout	
Sex	Male
Age (years)	65
Disease duration (years)	3
Treatment	
Nonsteroidal anti-inflammatory drug	

Data presented as *n *or median (range).

### Immunohistochemistry

Immunohistochemistry analyses were performed on formalin-fixed paraffin-embedded tissues. Polymorphonuclear cells and peripheral blood mononuclear cells, obtained as previously described [10], were injected into a fragment of a murine intestine. The intestine fragment was then processed as a tissue for immunohistochemistry. Stalk-1, Aprily-2 (and its competition with soluble APRIL), Aprily-8, anti-CD138, anti-human immunoglobulin, anti-elastase and the corresponding secondary antibodies have all been previously described [10]. The anti-CD68 (clone PG-M1, IgG_3_; Santa Cruz, CA, USA) was used at 3 μg/ml with anti-IgG_3 _conjugated to biotin and streptavidin conjugated to Alexa 488 (Becton Dickinson Biosciences, San Jose, CA, USA). The anti-heparan sulfate 10e4 (Seikagaku Corporation, Tokyo, Japan) was used at 1 μg/ml.

Images were visualized under light or fluorescent microscopy with Axiophot 1 (Carl Zeiss AG, Berlin, Deutschland), captured with an axiocam (Carl Zeiss AG) color CCD camera, and treated on a Pentium III computer with axioVision™ software (Carl Zeiss AG). The original magnification was 20× unless stated. In some experiments, 4',6'-diamidino-2-phénylindole staining was included in the merged images. Confocal analyses were performed with a LSM 510 microscope (Carl Zeiss AG). For quantification of the Aprily-2 signal, images of the lining layers and blood vessels for RA lesions and for normal synovium, respectively, were acquired with the 40× objective. An area of identical size in a zone exhibiting the strongest staining was selected for each lesion, and was processed using MetaMorph Image Analysis software (Molecular Devices, Union City, CA, USA). Color thresholds were selected in the Hue, Saturation, Intensity space. Saturation values obtained in the threshold field were added, logged to a spreadsheet, and expressed in arbitrary units. Stalk-1 stained cells were numerated in the corresponding area from a serial cut, after image acquisition with the 40× objective and counting the entire field, corresponding to a tissue area of 30 mm^2^.

### Flow cytometry

Human umbilical vein endothelial cells (HUVECs) were cultured as described by Bradfield and colleagues [28]. The cells were harvested from culture dishes with PBS 3 mM ethylenediamine tetraacetic acid at 37°C and were washed twice with PBS. Binding of ACRP30-APRIL in the presence or absence of heparin was performed as previously described [10]. Binding was assessed by flow cytometry on a FACSCAN and Cellquest (Becton Dickinson Biosciences).

## Results

### Presence of secreted APRIL in normal synovium

**Figure 1:**
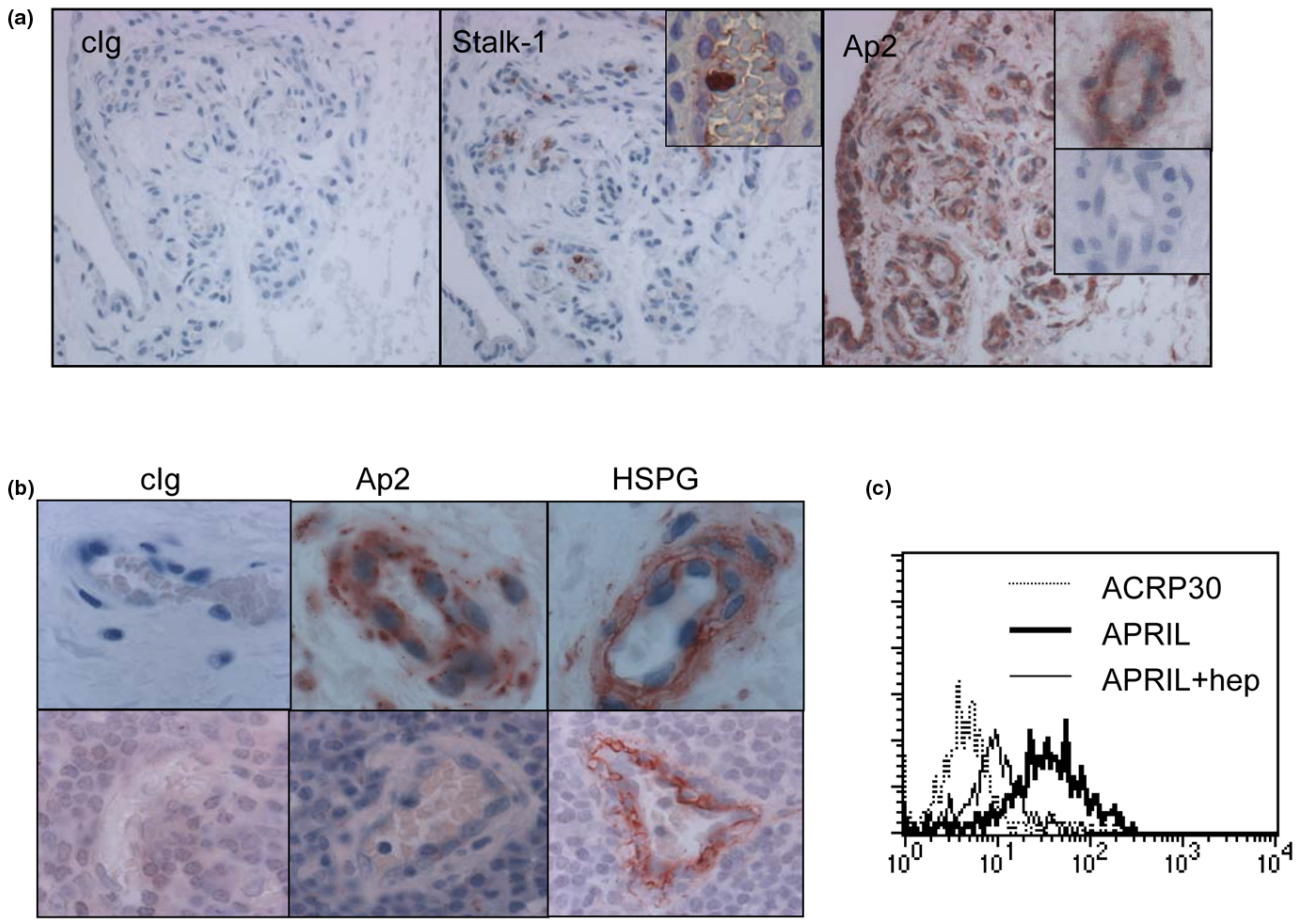
Concentration of secreted APRIL in normal synovium. **(a) **Normal synovium was immunostained with control immunoglobulin (cIg) (left panel) and Stalk-1 (middle panel). Stalk-1 stained cells are present in blood vessels (lower insert). A serial section of the same synovium was immunostained with Aprily-2 (right panel). Insert: Aprily-2 staining in the absence (upper) or presence of recombinant soluble A proliferation-inducing ligand (APRIL) (lower). **(b) **Normal synovium (upper panels, n = 4) and non-inflamed tonsils (lower panels, n = 7) were immunostained with cIg (left panel), Aprily-2 (middle panel), and anti-heparan sulfate proteoglycans (HSPG) (right panel). Blood vessels from these two organs are shown. Original magnification, 100×. **(c) **Binding of recombinant APRIL on the surface of human umbilical vein endothelial cells was assessed by flow cytometry in the presence or absence of heparin (hep). ACRP30 was used as a negative control.

We first assessed by immunohistochemistry the presence of APRIL-producing cells and secreted APRIL in normal synovial tissues. Cells producing APRIL, detected by the Stalk-1 antibody, were not present. In rare cases, positive cells were present in blood vessels irrigating the tissue (Figure 1a, insert, middle panel). Despite the paucity in APRIL-producing cells, the secreted product, detected by the Aprily-2 antibody, was significantly present in these samples, bound onto cells from the lining layer and endothelial cells (Figure 1a, right panel). We obtained a similar staining with Aprily-8, a second mAb against secreted APRIL (data not shown). This staining in normal synovium was specific, since it was abolished by preincubation of the anti-APRIL mAb with soluble APRIL (Figure 1a, right panel, inserts). Owing to their reactivity against the furin-processed extracellular domain of APRIL, we cannot exclude that Aprily-2 and Aprily-8 recognized the hybrid molecule between TNF-like weak inducer of apoptosis and APRIL, TWEPRIL [29], and heterotrimers between BAFF and APRIL [30] in these tissues.

The presence of secreted APRIL on synovial blood vessels correlated well with their expression of the APRIL-binding partner, HSPG (Figure 1b). We did not observe such accumulation of secreted APRIL in blood vessels irrigating a secondary lymphoid organ, such as tonsils, despite the fact that tonsil vessels expressed comparable amounts of HSPG. In an *in vitro *staining assay, we confirmed binding of secreted APRIL at the surface of endothelial cells (Figure 1c). In this experiment, APRIL bound to HSPG since it was inhibited by heparin, a potent antagonist of APRIL/HSPG interactions [12].

Taken together, these findings indicate that the normal synovial tissue concentrates specifically secreted APRIL.

### Blood neutrophils secrete constitutively APRIL

Most proteins in the synovium originate from the blood [31]. This process is due to the fenestration of blood vessels irrigating this tissue [32]. APRIL concentrated by the synovial tissue may therefore originate from blood. To test this hypothesis, we studied APRIL expression in blood.

**Figure 2:**
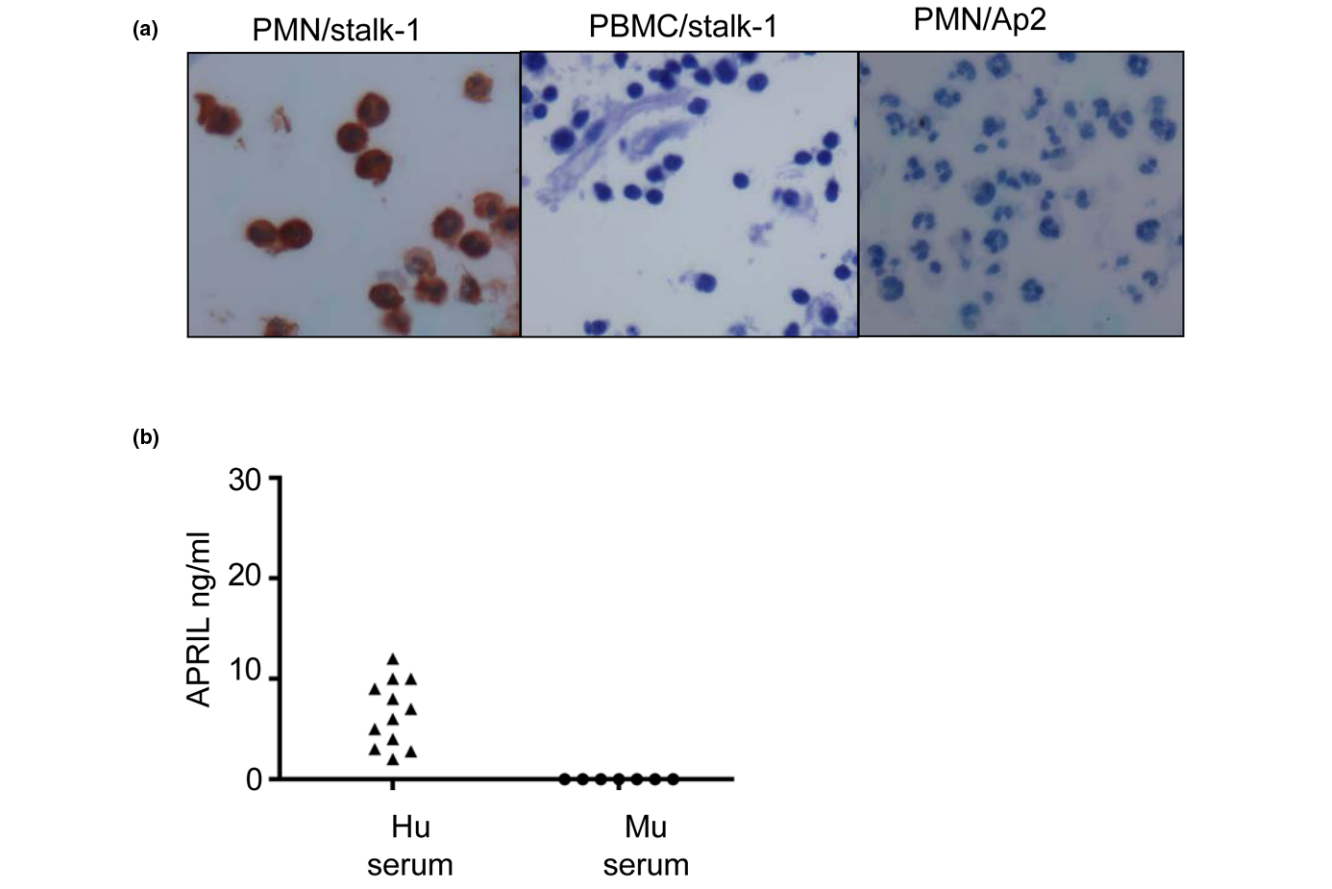
Blood neutrophils secrete constitutively APRIL. **(a) **Polymorphonuclear cells (PMN) and peripheral blood monoculear cells (PBMC) were embedded into mouse intestine and stained with Stalk-1 and Aprily-2. **(b) **Levels of A proliferation-inducing ligand (APRIL) in serum were determined with an ELISA KIT for human APRIL in healthy donors (Hu). To assess the specificity of the detection, mouse xenogeneic sera were tested as negative control (Mu).

We have already reported that blood neutrophils express APRIL mRNA in the steady state [10]. In the present article we further show that APRIL-producing cells were confined to the polymorphonuclear cell fraction, since these APRIL-producing cells were absent from the peripheral blood monoculear cell fraction. Blood polymorphonuclear cells readily secrete all of the APRIL they produce, since we observed no staining with Aprily-2 (Figure 2a). This constitutive secretion of APRIL by blood polymorphonuclear cells is consistent with the presence of secreted APRIL over the nanogram per milliliter range (median, 6.1 ng/ml; range 1 to 12 ng/ml) in sera from healthy donors (Figure 2b). This result is in accordance with previous studies [9,33], but is far inferior to the median of 96.7 ng/ml measured in RA sera with the same ELISA kit [34]. This detection we observed in normal sera is specific to APRIL, since it was not observed in xenogeneic murine sera. Secreted APRIL present in the synovium is therefore most probably diffusing from the blood, owing to the fenestration of synovial vessels.

### APRIL production and retention in inflamed RA synovium

**Figure 3:**
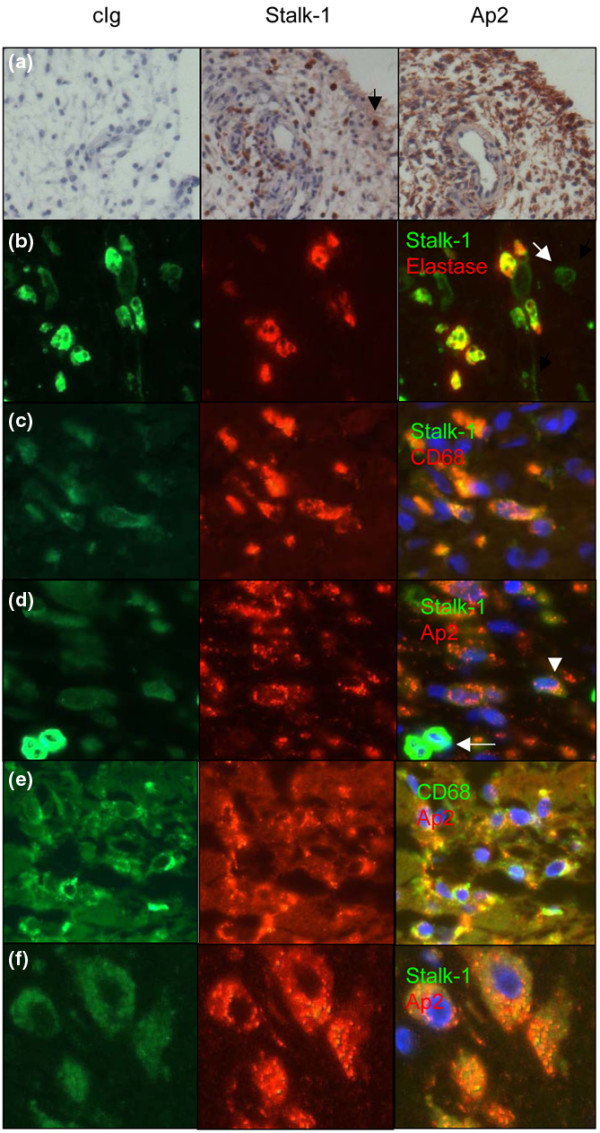
APRIL production increases in synovium from rheumatoid arthritis patients. **(a) **Control immunoglobulin (cIg), Stalk-1 and Aprily-2 stainings for a synovium from a rheumatoid arthritis (RA) patient. Arrow: Stalk-1 stained cell present in the lining layer. **(b) **Two-color immunofluorescence shows that Stalk-1^bright ^cells (green) are elastase^+^(red) neutrophils. Stalk-1^dull ^cells do not express elastase (arrow). **(c) **Stalk-1^dull ^cells (green) are macrophages expressing CD68 (red). **(d) **Stalk-1^dull ^cells produce A proliferation-inducing ligand (APRIL) (green) and are positive for secreted APRIL (red) (arrowhead), while Stalk-1^bright ^cells (arrow) do not. **(e) **CD68^+ ^macrophages (green) retain secreted APRIL (red). **(f) **Two-color immunofluorescence and confocal analysis shows that Stalk-1 staining (green) is associated with Aprily-2 (red) staining in a single cell, but is not fully colocalized. Original magnification, 100×. Pictures representative of 11 RA lesions.

We next analyzed inflamed synovial tissues from RA patients and non-RA patients. APRIL-producing cells were again present in blood vessels, but the majority was now infiltrating the synovial tissue or the lining layer in RA lesions (Figure 3a). The majority of the cells in the vicinity retained secreted APRIL. We identified APRIL-producing cells infiltrating the tissue as neutrophils expressing elastase in such inflamed tissues (Figure 3b). These neutrophils were all brightly stained with Stalk-1, but a second population - exhibiting a dull Stalk-1 staining and no expression of elastase - was also present. We identified these cells as macrophages expressing CD68 (Figure 3c). The intensity of Stalk-1 staining was not the only difference between APRIL-producing synovial neutrophils and macrophages. Indeed, Aprily-2 stained the Stalk-1^dull ^macrophages but not the Stalk-1^bright ^neutrophils (Figure 3d). Co-staining of CD68 with Aprily-2 confirmed that the dull Aprily-2^+ ^cells were synovial macrophages (Figure 3e). Confocal analysis showed that the overlap between Stalk-1 and Aprily-2 staining was not complete in the Stalk-1^dull ^cells (Figure 3f). This indicates that synovial macrophages retained the secreted APRIL they produced, and/or bound secreted APRIL from the extracellular medium.

Aprily-2 also stained Stalk-1^-^ cells, indicating that nonproducing cells also retained secreted APRIL. Stalk-1^+ ^cells were also present in the lining layer from a minority (3/9 patients) of RA patients. The lining layer contains fibroblast-like synoviocytes, reported by others to produce APRIL when cultured [23]. The Stalk-1 stained cells in the lining layer, however, were not fibroblast-like synoviocytes based on morphological criteria. T cells can also produce APRIL [29], but the T cells infiltrating RA lesions were not stained by Stalk-1 (data not shown).

**Figure 4:**
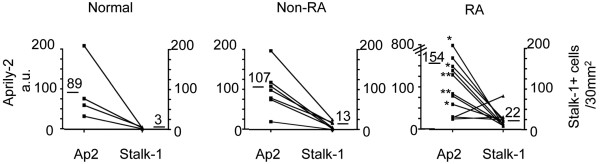
Quantification of APRIL production and secretion in healthy and rheumatoid arthritis synovium. Quantification of the Aprily-2 staining (left *y *axis) and the number of Stalk-1-stained cells (right *y *axis) for normal synovial tissues (n = 4), for non-rheumatoid arthritis (RA) lesions (n = 7) and for RA lesions (n = 11). Arbitrary units are used for Aprily-2 quantification. Number of stalk-1-stained cells shown for an adjacent area of 30 mm^2^. Mean values are indicated. *RA lesions with germinal centers and plasma cells. **Seronegative RA lesions. APRIL, A proliferation-inducing ligand.

We noticed that APRIL expression was variable among patients with the same disease entity, but was very similar when non-RA patients and RA patients were compared. Figure 4 provides a quantification of secreted APRIL in selected zones exhibiting the highest retention of APRIL from normal synovium, non-RA lesions and RA lesions. Secreted APRIL expression per surface of tissue increased by only 1.4-fold and 1.7-fold upon synovium inflammation in non-RA and RA patients, respectively, despite the fact that the number of cells producing APRIL increased by more than fourfold and sevenfold, respectively. The lesions from patients with Lyme's disease had the lowest number of APRIL-producing cells and secreted APRIL. The seronegative RA cases were not different from the seropositive cases in this analysis. In these inflamed lesions, we again observed zones with high concentration of secreted APRIL and a low number of producing cells in the adjacent area, confirming that the synovium efficiently retains secreted APRIL.

Taken together, inflammatory reactions within synovial tissues recruit neutrophils and macrophages, insuring high levels of APRIL production, both in non-RA lesions and RA lesions. Retention of secreted APRIL per zone was only slightly increased compared with normal synovium.

### Plasma cells in RA lesions accumulate in APRIL-rich niches

**Figure 5:**
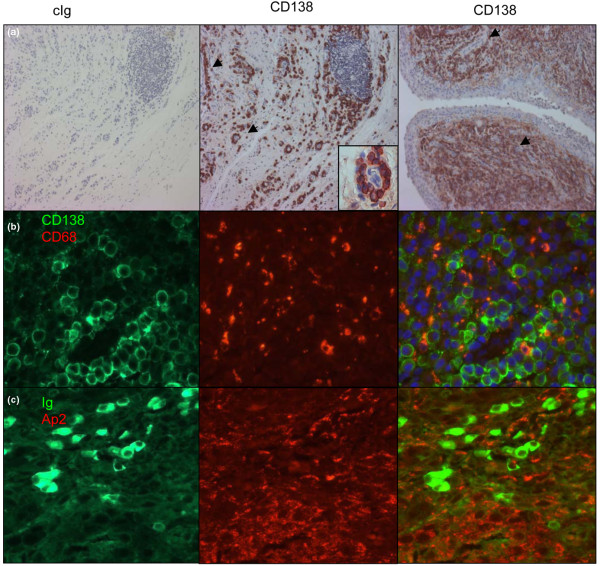
Synovial plasma cells in rheumatoid arthritis lesions accumulate in zone of secreted APRIL retention. **(a) **A rheumatoid arthritis (RA) lesion was stained with a control immunoglobulin (cIg) or an anti-CD138. Middle panel: zone with a germinal center (GC) and a low CD138^+ ^plasma cell (PC) density; arrows indicate examples of the many blood vessels harboring PC accumulation; Insert, high magnification of PCs accumulating around a blood vessel. Right panel: same RA lesion in an area with high CD138^+ ^PC density; arrow indicates example of PCs accumulating around blood vessels. **(b) **CD138^+ ^plasma cells (green) at high density are in close contacts with CD68^+ ^macrophages (red). **(c) **Ig^+ ^PCs (green) at high density accumulate close to secreted A proliferation-inducing ligand (APRIL) (red). Pictures are representative of three RA lesions with ectopic GC formation and PC accumulation.

We next studied PC localization in RA synovial tissues with ectopic GCs. One should note that such RA lesions did not show significantly more APRIL expression that non-RA lesions or RA lesions without GCs (see Figure 4). CD138^+ ^PCs localized in the periphery of GCs (Figure 5). The GC periphery, however, was a transit area for synovial PCs, since we observed them mostly concentrated around blood vessels, forming a full crown in the case of small vessels (insert). When the density of PCs became high, they were still around blood vessels but were also extending towards the lining layer. At high density, PCs were in close contact with CD68 macrophages (Figure 5a), in zones of high concentration of secreted APRIL (Figure 5b). PCs therefore accumulate in APRIL-rich zones of highly inflamed synovial tissues.

## Discussion

In the present study we report a significant expression of APRIL in the synovium of normal donors, non-RA and RA patients. Concentration of secreted APRIL per defined area was only slightly upregulated in non-RA and RA patients compared with normal donors. In contrast, the density of APRIL-producing cells greatly increased in inflamed synovium. Recruitment of APRIL-producing cells is therefore not specific to RA, but the presence of such cells in non-RA lesions is consistent with the detection of secreted APRIL in the synovial fluid from non-RA patients reported by others [35,36]. Since neutrophils are the major source of APRIL in these latter lesions, our study indicates that presence of APRIL is a consequence of the ongoing inflammation in these diseases. In non-RA lesions, APRIL may be a bystander inflammatory product with no specific role in the pathological process, owing to the current knowledge in APRIL physiology.

Since APRIL plays a major role in the immunoglobulin switch process and PC survival, one may expect that APRIL promotes autoimmune reactions associated with production of autoantibodies, such as RA. In the present study we report the presence of cells producing and secreting APRIL, including neutrophils and macrophages within RA lesions. Neutrophils constitute a major source of APRIL in tissues, while macrophages generally do not produce this molecule [37]. Synovial macrophages are therefore different from macrophages in other tissues. In addition, synovial macrophages are also different from neutrophils in their mode of APRIL production. They produce less APRIL, and process efficiently the full-length product - but instead of secreting all of the produced APRIL, they retain some of it and/or bind secreted APRIL present in the extracellular medium. In contrast, retention and binding of secreted APRIL by neutrophils has never been observed. This is probably due to the lack of HSPG expression by neutrophils [10], compared with macrophages [38].

We frequently noticed a discrepancy between the concentration of the secreted APRIL product within the tissues and the density of APRIL-producing cells. This difference was best observed in normal synovium. Indeed, in samples from subjects without arthritis, we detected significant levels of secreted APRIL, covering the lining layer and endothelial cells, despite the paucity of APRIL-producing cells. This finding indicates that the normal synovial tissue is able to retain substantial amounts of secreted APRIL. Blood vessels irrigating the synovium are fenestrated, allowing the diffusion of blood proteins, and it is probable that APRIL produced constitutively by circulating neutrophils diffuses from the blood to accumulate into the synovial tissue. We previously showed that HSPG retain APRIL in various tissues [8,10,39,40]. In a healthy synovium, syndecan-3/glypican-4 and syndecan-2/syndecan-3/glypican-4 - expressed by the lining layer and endothelial cells, respectively [41] - are likely to be the proteic carriers of heparan sulfate chains mediating APRIL retention. The accumulation of APRIL by the synovial vessels is specific to this tissue, since tonsil vessels do not retain secreted APRIL, consistent with the absence of fenestration for blood vessels in tonsils.

The study of lesions with ectopic GCs and generation of PCs led to some valuable observations regarding the role of APRIL in RA. Indeed, we noticed that PCs migrate from GCs towards endothelial cells. PC localization around blood vessels in RA lesions was reported a long time ago [42]. The recent detection of the PC chemoattractant CXCL-12 on synovial vessels explained this homing [43]. In the present study, we further provide evidence that PCs receive a survival signal from these endothelial cells, given in a trans fashion, similarly to tonsillar epithelial cells [8]. When PCs accumulate extensively, they were also in contact with cells from the lining layer and synovial macrophages, both rich in secreted APRIL. The accumulation of APRIL in several synovial cells may therefore explain the persistence of PCs within the inflamed synovial tissue, as already proposed [27].

## Conclusions

The present study demonstrates that normal synovium and pathologic synovium both for non-RA and RA patients retain soluble APRIL, constituting APRIL-rich niches. These niches are similar to those recently observed in mucosa-associated lymphoid tissues, wherein plasma cells survive to secrete locally antibodies against infectious agents. In non-RA lesions, these niches may not be functional. These APRIL-rich niches may provide an adequate environment for synovial PCs in RA lesions, therefore contributing to the generation of pathogenic autoantibodies. The specific retention of soluble APRIL in a normal synovium indicates that this organ offers a favorable environment for PCs, even before disease onset.

## Abbreviations

APRIL: A proliferation-inducing ligand; BAFF: B-cell activation factor; BCMA: B-cell maturation antigen; GC: germinal center; HSPG: heparan sulfate proteoglycans; mAb: monoclonal antibody; PBS: phosphate-buffered saline; PC: plasma cell; RA: rheumatoid arthritis; TACI: transmembrane activator, calcium modulator and cyclophilin ligand interactor; TNF: tumor necrosis factor.

## Competing interests

The authors declare that they have no competing interests.

## Authors' contributions

CG, VK, CAS and CC provided human samples and analyzed the data. CG and CC wrote the manuscript. CB performed research. BH designed the study, performed the research, analyzed the data and wrote the manuscript.
